# Mechanistic evaluation of long-term in-stent restenosis based on models of tissue damage and growth

**DOI:** 10.1007/s10237-019-01279-2

**Published:** 2020-01-07

**Authors:** Ran He, Liguo Zhao, Vadim V. Silberschmidt, Yang Liu

**Affiliations:** Wolfson School of Mechanical, Electrical, and Manufacturing Engineering, Loughborough University, Epinal Way, Loughborough, LE11 3TU UK

**Keywords:** Finite element, In-stent restenosis, Stent deployment, Arterial damage, Tissue-growth model

## Abstract

Development and application of advanced mechanical models of soft tissues and their growth represent one of the main directions in modern mechanics of solids. Such models are increasingly used to deal with complex biomedical problems. Prediction of in-stent restenosis for patients treated with coronary stents remains a highly challenging task. Using a finite element method, this paper presents a mechanistic approach to evaluate the development of in-stent restenosis in an artery following stent implantation. Hyperelastic models with damage, verified with experimental results, are used to describe the level of tissue damage in arterial layers and plaque caused by such intervention. A tissue-growth model, associated with vessel damage, is adopted to describe the growth behaviour of a media layer after stent implantation. Narrowing of lumen diameter with time is used to quantify the development of in-stent restenosis in the vessel after stenting. It is demonstrated that stent designs and materials strongly affect the stenting-induced damage in the media layer and the subsequent development of in-stent restenosis. The larger the artery expansion achieved during balloon inflation, the higher the damage introduced to the media layer, leading to an increased level of in-stent restenosis. In addition, the development of in-stent restenosis is directly correlated with the artery expansion during the stent deployment. The correlation is further used to predict the effect of a complex clinical procedure, such as stent overlapping, on the level of in-stent restenosis developed after percutaneous coronary intervention.

## Introduction

Atherosclerosis is a progressive vascular disease, resulting in the narrowing of lumen, compared to its healthy condition, due to the build-up of plaque inside an artery wall (Libby 2002). The plaque development is caused by migration and proliferation of native cells and local accumulation of blood-borne species, which include lipid, fibro-fatty composites and calcium salts (Singh et al. 2002). Percutaneous coronary intervention (PCI) is a minimally invasive surgical procedure to treat atherosclerotic arteries. The development of PCI started with angioplasty, for which a small balloon, inserted to the diseased part of an artery, is used to open the blocked artery through inflation. However, an acute vessel recoil often occurs after angioplasty. To eliminate the problem, stents, either balloon- or self-expandable, were developed and introduced to PCI, aiming to support the artery after expansion. The development of stents began with bare-metal stents (BMSs) and then progressed to drug-eluting stents (DESs) and bioresorbable vascular scaffolds (BVSs). BMSs, made of pure metals (no coating), were found to associate with a high occurrence of neointimal hyperplasia—enlargement of internal arterial layer—after implantation, leading to a re-narrowing of the treated artery, i.e., in-stent restenosis (ISR). To reduce the rate of ISR, polymer coatings, loaded with drugs that could inhibit neointimal growth, were introduced to cover the metal struts, leading to the revolutionary development of DESs. Recently, BVSs, made of biodegradable polymers or metals, were developed for complete bioresorption after the implantation, with the hope of further reducing ISR as well as late stent thrombosis caused by the permanent presence of metallic stents.

ISR is one of the major issues for PCI and defined as a decrease in luminal diameter by more than 50% after stent implantation (Hamid and Coltart 2007). The incidence of ISR (binary restenosis rate) for BMSs was found to be between 20 and 30%, based on medium- and long-term follow-ups of clinical trials (Iqbal et al. 2013). DESs are associated with a significantly reduced rate of ISR (% diameter stenosis (DS)) compared to BMSs. For example, the SPIRIT I trial showed that the rate and incidence of ISR for XIENCE V DESs were 16% and 0%, respectively, significantly lower than those (39% and 25.9%, respectively) for BMSs (Serruys et al. 2005). In ENDEAVOR II trial, the Endeavor DESs also demonstrated better outcomes than BMSs, in terms of the rate (27.8% for Endeavor vs. 42.3% for BMS) and incidence (9.5% for Endeavor vs. 33.5% for BMS) of ISR (Kandzari and Leon 2006). For BVSs, the ABSORB GT1™ (Abbott Vascular, USA) was the first, and also the only, stent that received both the European CE marking and US Food and Drug Administration approval. However, several independent randomized controlled trials (RCTs) showed the ABSORB BVSs caused a higher ISR rate than DESs (Rizik et al. 2017), including ABSORB China (18.5% for ABSORB vs. 11.3% for DES), ABSORB Japan (17.4% for ABSORB vs. 11.7% for DES), EVERBIO II (16.9% for ABSORB vs. 11.3% for DES) and TROFI II trials (17.3% for ABSORB vs. 14.5% for DES). In addition to stent technology, the procedure-related factors also pose a risk to the development of ISR, such as the number and length of stents being used and stent overlapping. Specifically, the implantation of multiple, longer and overlapped stents increased the occurrence of ISR (Hoffmann and Mintz 2000).

A brief description of the structure of, and the biological response to tissue damage in, arteries is given below to support the development of the mechanical model of restenosis. A healthy arterial wall has three distinct layers, i.e., the intima, media and adventitia layers. The intima consists of one layer of endothelial cells, a subendothelial layer and an internal elastic lamina supporting the endothelium (Crawford et al. 2009). The media, the middle layer of the artery wall, is made of smooth muscle cells (SMCs) and elastic collagen fibrils. The adventitia, the outermost layer of the artery wall, is mainly composed of fibroblasts and fibrocytes, a histological ground substance and thick bundles of collagen fibrils forming a fibrous tissue. During the development of atherosclerosis, plaque forms in the intima layer and builds up between the endothelium and the media layer. As stent is in direct contact with plaque during the PCI procedure, it causes damage and rupture to the plaque as a result of expansion. On the other hand, mechanical stretching of the vessel wall also induces damage and injury to the media and adventitia layers, activating the transmigration of the leucocyte into the vessel wall. This process triggers a proliferation of SMCs in the media layer, which then migrates to form neointima and thicken the vessel wall (Hoffmann and Mintz 2000). Although the inflammatory events begin as beneficial wound-healing responses, an adverse vascular change happens in the end, leading to re-narrowing of the blood vessel, or ISR (Bennett and O’Sullivan 2001; Evans et al. 2008).

Hence, a correlation between the stenting-induced vessel injury and the rate of ISR has been investigated with patient and animal studies. For instance, stents were implanted into coronary arteries of pigs to examine the contributions of stenting-induced arterial injury and inflammatory response to neointimal hyperplasia (Schwartz et al. 1992; Kornowski et al. 1998). A level of arterial injury was found positively correlated with the inflammatory reaction and neointimal formation. In fact, monocytes, a type of leucocyte, were observed in rabbits implanted with endovascular metal stents. They adhered to the inner surface of stent-injured arteries and then penetrated the arterial wall, resulting in neointima formation (Rogers et al. 1996). Also, Farb et al. (2002) examined 87 stented coronary arteries taken from 56 patients and showed that medial damage induced by coronary stenting increased arterial inflammation, associated with an increased neointimal growth. Furthermore, Dussaillant et al. (1995) used volumetric intravascular ultrasound to assess the ISR in 44 patients treated with Palmaz-Schatz stents. It was found that the volume of neointimal hyperplasia was greater in restenotic stents, and the pattern of ISR was related to the distribution of neointimal tissue.

Therefore, modelling-based studies were also attempted to correlate quantitatively the arterial damage and the ISR. For instance, agent-based model (ABM) has been used to simulate the SMC growth in response to the stent-induced damage obtained from FE analysis (Zahedmanesh et al. 2014; Nolan and Lally 2018). The model demonstrated a direct correlation between the stent deployment diameter and the level of ISR. Coupled with FE analysis, the ABM was adopted by Keshavarzian et al. (2018) to model the response of vessels to the levels of growth factors, proteases, signalling molecules and blood pressure. The ABM was also applied for multiscale modelling of stent deployment, and subsequent blood flow and tissue growth in a stented vessel (Tahir et al. 2013, 2015; Zun et al. 2019, 2017), in agreement with in vivo post-stenting data. On the other hand, Holzapfel et al. (2000, 2002) developed a three-dimensional model for simulation of balloon angioplasty and stent deployment in a stenotic artery to gain a mechanistic understanding of the PCI procedure. Their results showed that stresses in a vessel wall were significantly localized and component-specific, which might activate the migration and proliferation of SMCs and subsequently led to ISR. Lally and Prendergast (2006) used a damage-adaptive finite element (FE) approach to simulate ISR for three different stent designs. Their results for ISR demonstrated that the restenotic growth was concentrated around the stent struts and greatest at the ends of the stents. However, plaque was not considered in their simulations, while the arterial wall was modelled as a hyperelastic isotropic material, representing a critical limitation. Very recently, Fereidoonnezhad et al. (2017) proposed a continuum tissue-growth model, which involved cellular changes in the arterial wall due to damage, for simulation of restenosis. They implemented the model into the FE software Abaqus via a user-defined material subroutine (UMAT), which was verified against the results obtained from a MATLAB code. Then, the model was applied to simulate the development of restenosis after angioplasty, and the results matched clinical observations qualitatively. Cheng and Zhang (2019) presented a growth model similar to the one proposed by Fereidoonnezhad et al. (2017), but its growth factor was linked to the stress instead of the damage. The model was also used to simulate the arterial wall remodelling after stenting, using the FE method. Their results suggested that the volumetric growth of the artery wall tended to even out, lowering both the overall stress and the stress concentration.

The correlation between stenting-induced vessel injury and the rate of ISR has been investigated in the literature, but the findings relied only on empirical data for certain target vessels and stent types. These data were generated by either (1) measuring the lumen loss of stented arteries through intravascular ultrasound imaging (Hoffmann et al. 2002) and autoradiography (Clowes et al. 1989) or (2) defining an injury score based on histological sections which was correlated with the neointimal growth (Schwartz et al. 1992). However, so far, it was unable to correlate damage mechanisms (including property and morphology changes) inside the arterial tissue with the load condition during and after PCI. Therefore, the relationship between the stress inside the vascular tissue and the resulting ISR is still not known. The contact pressure between the balloon/stent and artery causes vascular damage, manifested in the form of tissue softening, fibre rupture/reorientation, cell migration and proliferation and endothelial denudation. The harmful contact pressure depends on the area of the stent surface, the final diameter of the stent, expansion mechanisms of the stent and balloon, and the pressure inside the balloon. Clearly, the quantification of vascular damage and ISR based on mechanical data has not yet been published, and this is a limitation of existing studies in modelling of vascular growth after PCI with stenting.

The aim of this paper is to investigate the contribution of tissue damage in the media layer to ISR after stent deployment, with a focus on predicting the development of ISR with time. In the current study, appropriate hyperelastic models were adopted to describe the damage of plaque and arterial layers caused by stent deployment. A tissue-growth model, associated with vessel damage, was introduced to predict the growth of the media layer, a major contributor to ISR. Systematic FE simulations were carried out to analyse the stenting procedure, quantify the tissue damage caused by stenting and predict the subsequent evolution of ISR over a defined time. In addition, different commercial stents were explored further to study the effects of stent design and material on ISR, and, more importantly, to establish a direct correlation between artery expansion and the rate of ISR. The correlation was then used to predict the evolution of ISR caused by stent overlapping, a more complex clinical procedure. The novelty of this paper includes: (1) assessment of stenting-induced arterial damage during PCI, (2) prediction of ISR after PCI and (3) correlation of ISR with stenting-induced arterial damage. In particular, the diseased artery was described by one of the latest anisotropic hyperelastic and tissue damage models during the simulations of PCI.

## Constitutive models

To capture the complex mechanical behaviours of tissue components, a series of models was established to describe its constitutive behaviours together with the material properties of stent and balloon, used as inflate devices.

### Hyperelastic model with damage for plaque

The first-order Ogden hyperelastic model (Ogden 1972) with Mullins effect (i.e., stress-softening or damage; Ogden and Roxburgh 1999) was used to describe the constitutive behaviour of the plaque (assumed to be isotropic), for which the pseudo-energy potential is given as:

1 $${\psi = \psi_{vol} + \eta \bar{\psi }^{0} + \phi \left( \eta \right) = \frac{1}{D}\left( {J - 1} \right)^{2} + \eta \frac{2\mu }{{\alpha^{2} }}\left( {\bar{\lambda }_{1}^{\alpha } + \bar{\lambda }_{2}^{\alpha } + \bar{\lambda }_{3}^{\alpha } - 3} \right) + \phi \left( \eta \right),}$$

where $\psi_{{\text{vol}}}$ and $\bar{\psi }^{0}$ are the volumetric and isochoric parts of strain energy, respectively, $\phi \left( \eta \right)$ is the smooth discontinuous damage function, $\mu$, $\alpha$ and $D$ are the material parameters, $J$ is the volumetric ratio, $\lambda_{1}$, $\lambda_{2}$ and $\lambda_{3}$ are the principal stretches (ratios), the bar denotes isochoric values and the superscript 0 refers to the primary loading path. In addition, $\eta \in \left[ {0,1} \right]$ is the damage variable expressed as:

2 $$\eta = 1 - \frac{1}{r}{\text{erf}}\left[ {\frac{1}{m}\left( {\bar{\psi }^{\hbox{max} } - \bar{\psi }^{0} } \right)} \right],$$

where $m$ and $r$ are the positive parameters, ${\text{erf}}\left( {} \right)$ is the error function and $\bar{\psi }^{\hbox{max} }$ denotes the maximum strain energy in the deformation history.

**Fig. 1:**
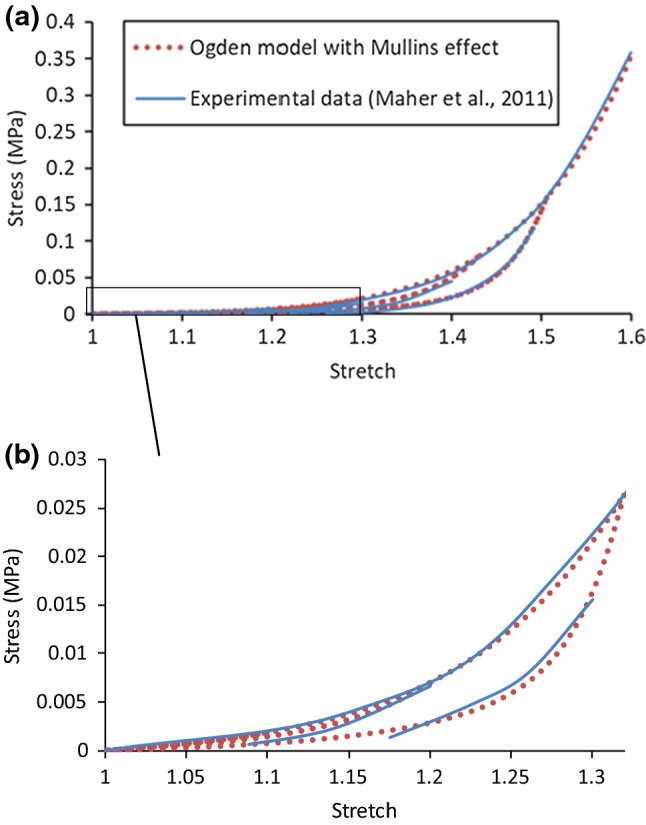
**a** Stress-stretch response of plaque simulated using Ogden model with Mullins effect, in comparison with experimental data for echolucent plaque in Maher et al. (2011) (unloading occurred at stretch levels of about 1.2, 1.3, 1.4 and 1.5); **b** a zoomed-in view of the low strain region

The model parameters were determined by fitting the experimental stress-stretch data obtained by Maher et al. (2011) for echolucent plaque of human carotid artery, and the corresponding values are given in Table 1, where *ρ* is the density of the plaque with value given in Rahdert et al. (1999). The stress-stretch response for the plaque, simulated under the uniaxial tension, is shown in Fig. 1, demonstrating a good agreement with the experimental results (Maher et al. 2011).

**Table 1:** Parameter values of Ogden model with Mullins effect for plaque

*ρ* (t/mm^3^)	$\mu$ (MPa)	$\alpha$	$D$ (MPa^−1^)	*r*	*m* (mJ/mm^3^)
1.22E−9	0.00396803	13.8367	0.239019	1.3	0.008

### Hyperelastic model with damage for arterial layers

The modified HGO-C model (Nolan et al. 2014) with damage (Fereidoonnezhad et al. 2016) was used to describe the anisotropic hyperelastic constitutive behaviour of arterial layers, with a pseudo-energy potential given by

3 $$\begin{aligned} \psi = \psi_{vol} + \bar{\psi }_{m}^{0} + \mathop \sum \limits_{\alpha = 1}^{N} \left[ {\eta_{f,\alpha } \psi_{f,\alpha }^{0} + \phi_{f,\alpha } \left( {\eta_{f,\alpha } } \right)} \right] - \left[ {\left( {1 - \eta_{in} } \right)\psi_{in}^{*} \left( {I_{i}^{ *} } \right) + \phi_{in} \left( {\eta_{in} } \right)} \right], \end{aligned}$$

4 $$\begin{aligned} \psi_{{\text{vol}}} = \frac{1}{D}\left( {\frac{{J^{2} - 1}}{2} - \ln J} \right), \end{aligned}$$

5 $$\begin{aligned} \bar{\psi }_{m}^{0} = C_{10} \left( {\bar{I}_{1} - 3} \right), \end{aligned}$$

6 $$\begin{aligned} \psi_{f,\alpha }^{0} = \frac{{k_{1} }}{{2k_{2} }}\mathop \sum \limits_{\alpha = 1}^{N} \left[ {\exp \left( {k_{2} \left< \kappa \left( {\bar{I}_{1} - 3} \right) + \left( {1 - 3\kappa } \right)\left[ {I_{{4\left( {\alpha \alpha } \right)}} - 1} \right]\right>^{2} } \right) - 1} \right], \end{aligned}$$

where $\bar{\psi }_{m}$ and $\psi_{f,\alpha }^{0}$ are the isochoric energy stored in non-collagenous matrix and collagen fibres, respectively, $\phi_{f,\alpha } \left( {\eta_{f,\alpha } } \right)$ and $\phi_{in} \left( {\eta_{in} } \right)$ are the damage functions for the Mullins effect and permanent deformation, respectively, $C_{10}$ and $k_{1}$ are the stress-like parameters, $k_{2}$ is the dimensionless parameter, 〈 〉 stands for the Macaulay brackets, $\kappa \left( {0 \le \kappa \le 1/3} \right)$ is the temperature-dependent material parameter describing the level of dispersion in the fibre directions, *N* is the number of families of fibres (*N *≤ 3), $I_{1}$ is the first principal invariant of the right Cauchy–Green deformation tensor **C** (i.e., $I_{1} = {\text{tr}}{\mathbf{C}} = \lambda_{1}^{2} + \lambda_{2}^{2} + \lambda_{3}^{2}$ and its isochoric part is $\bar{I}_{1} = J^{ - 2/3} I_{1}$), $I_{{4\left( {\alpha \alpha } \right)}}$ are the invariants of **C** and ${\mathbf{a}}_{\alpha }$ (i.e., $I_{{4\left( {\alpha \alpha } \right)}} = {\mathbf{a}}_{\alpha } \cdot {\mathbf{Ca}}_{\alpha }$, with ${\mathbf{a}}_{\alpha }$ being the unit vectors used to define the mean directions of the fibres in the reference configuration). In addition, $\eta_{f,\alpha }$ and $\eta_{in}$ are the damage variables for the Mullins effect and permanent deformation, respectively, and $\psi_{in}^{*} \left( {I_{i}^{*} } \right)$ is the (anisotropic) inelastic energy dissipation, which are given by:

7 $$\begin{aligned} \eta_{f,\alpha } = 1 - \frac{1}{{r_{f} }}{\text{erf}}\left[ {\frac{1}{{m_{f} }}\left( {\psi_{f,\alpha }^{ \hbox{max} } - \psi_{f,\alpha }^{0} } \right)} \right], \end{aligned}$$

8 $$\begin{aligned} \eta_{in} = \frac{{\tanh \left[ {\frac{{\bar{\psi }_{m}^{0} + \psi_{f,\alpha }^{0} }}{{\left( {\bar{\psi }_{m} + \psi_{f,\alpha } } \right)^{max} }}} \right]^{{m_{2} }} }}{\tanh 1}, \end{aligned}$$

9 $$\begin{aligned} \psi_{in}^{*} \left( {I_{i}^{ *} } \right) = C_{10}^{ *} \left( {\bar{I}_{1}^{ *} - 3} \right) + \frac{{k_{1}^{ *} }}{{2k_{2}^{ *} }}\mathop \sum \limits_{\alpha = 1}^{\text{N}} \left[ {\exp \left( {k_{2}^{ *} \left<\kappa^{ *} \left( {\bar{I}_{1}^{ *} - 3} \right) + \left( {1 - 3\kappa^{ *} } \right)\left[ {I_{{4\left( {\alpha \alpha } \right)}}^{ *} - 1} \right] \right>^{2} } \right) - 1} \right], \end{aligned}$$

where $C_{10}^{*}$, $k_{1}^{*}$, $k_{2}^{*}$ and $\kappa^{*}$ are the material parameters for permanent deformation, and $\bar{I}_{1}^{*}$ and $I_{{4\left( {\alpha \alpha } \right)}}^{ *}$ are the strain invariants at the peak deformation of the loading history (i.e., $\bar{\psi }_{m}^{0} + \psi_{f,\alpha }^{0} = \left( {\bar{\psi }_{m} + \psi_{f,\alpha } } \right)^{\hbox{max} }$).

**Fig. 2:**
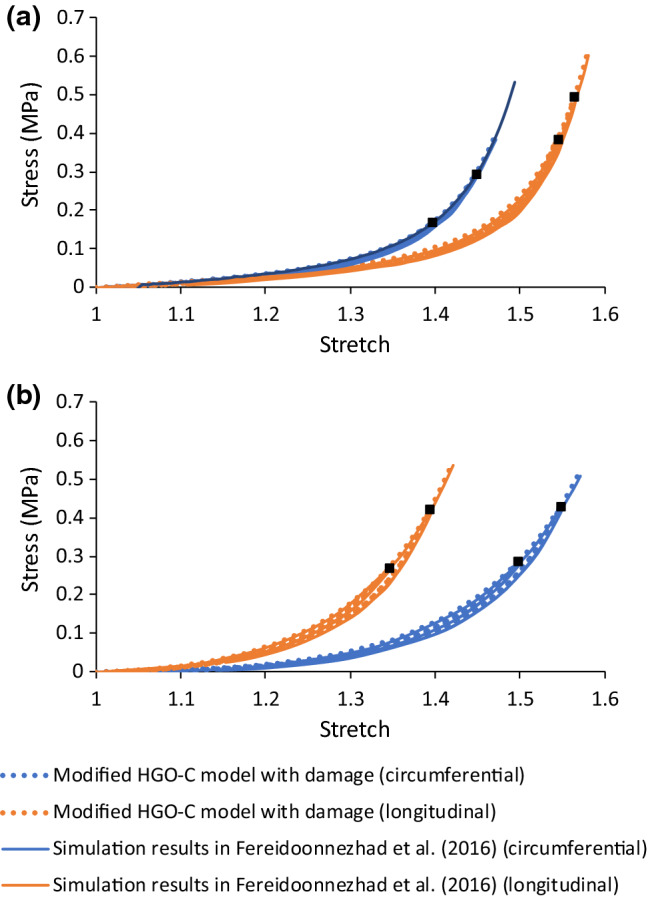
Stress-stretch responses of media (**a**) and adventitia (**b**) simulated using modified HGO-C model with damage, in comparison with those for thoracic aortas in Fereidoonnezhad et al. (2016) (black squares indicate unloading points)

Again, the model parameters were determined by fitting the experimental stress-stretch data for thoracic aortas (Weisbecker et al. 2012; Fereidoonnezhad et al. 2016). In this study, it was assumed that there were two families of fibres, embedded symmetrically in the tangential surface of each arterial layer, with $\varphi$ representing the angle between the mean direction of fibres and the circumferential direction in the artery. The fitted parameter values are given in Table 2 for the media and the adventitia layers. A VUMAT subroutine, interfaced with Abaqus, was written for the modified HGO-C model with damage. The stress-stretch responses for the arterial layers, also simulated under uniaxial tension, are shown in Fig. 2, which are in good agreement with the corresponding results in Fereidoonnezhad et al. (2016).

**Table 2:** Parameter values of modified HGO-C model with damage for arterial layers (Fereidoonnezhad et al. 2016)

Media						
*ρ* (t/mm^3^)	$C_{10}$ (MPa)	$D$ (MPa^−1^)	$k_{1}$ (MPa)	$k_{2}$	$\kappa$	$\varphi$ (°)
1.066E−9	0.020	0.001	0.112	20.610	0.24	41.0

$C_{10}^{ *}$ (MPa)	$k_{1}^{ *}$ (MPa)	$k_{2}^{ *}$	$\kappa^{ *}$	$r_{f}$	$m_{f}$ (MPa)	$m_{2}$
0.000529	0.001648	0.028	0.27	3.36	0.0151	3.03

Adventitia						
*ρ* *(*t/mm^3^)	$C_{10}$ (MPa)	$D$ (MPa^−1^)	$k_{1}$ (MPa)	$k_{2}$	$\kappa$	$\varphi$ (°)
1.066E−9	0.008	0.001	0.362	7.089	0.17	50.1

$C_{10}^{ *}$ (MPa)	$k_{1}^{ *}$ (MPa)	$k_{2}^{ *}$	$\kappa^{ *}$	$r_{f}$	$m_{f}$ (MPa)	$m_{2}$
0.000333	0.001445	0.460	0.27	2.70	0.0200	2.23

### Growth model for media

The growth model used for simulating the neointima formation was adopted from Fereidoonnezhad et al. (2017), based on a consideration of an intermediate configuration between the reference and the current ones. Since the neointima formation is associated with the inflammatory reaction to the stenting-caused damage in the media (Farb et al. 2002), the growth model was only applied to the media in the simulations. The deformation gradient **F** is multiplicatively decomposed into an elastic part ${\mathbf{F}}_{e}$ and a growth part ${\mathbf{F}}_{g}$:

10 $$\begin{aligned}\mathbf F = {\mathbf{F}}_{e} {\mathbf{F}}_{g} . \end{aligned}$$

Substituting Eq. (10) into ${\boldsymbol{\sigma}} = \frac{1}{J}\frac{\partial \psi }{{\partial {\mathbf{F}}}} \cdot {\mathbf{F}}^{\text{T}}$, we have

11 $$\begin{aligned}\boldsymbol \sigma = {\mathbf{F}}_{g}^{ - 1}\boldsymbol{\sigma}_{e} {\mathbf{F}}_{g}^{ - T} . \end{aligned}$$

A specific form is required for the growth tensor ${\mathbf{F}}_{g}$ in order to fulfil the constitutive formulation. The growth of the anisotropic arterial tissue is expected to be anisotropic; however, as no experimental data are available to characterize the anisotropic neointimal growth, an isotropic growth tensor was adopted here and is expressed as:

12 $$\begin{aligned} {\mathbf{F}}_{g} = \lambda_{g} {\mathbf{I}}, \end{aligned}$$

where $\lambda_{g}$ is the growth stretch.

Substituting Eq. (12) into Eq. (11) leads to

13 $$\begin{aligned}{\boldsymbol{\sigma}} ={\boldsymbol{\sigma}}_{e} / \lambda_{g}^{2} . \end{aligned}$$

To associate with the damage parameter $D_{g}$ in the modified HGO-C damage model introduced in Sect. 2.2, the growth stretch is proposed as (similar to the work of Fereidoonnezhad et al. (2017) and satisfies the continuity equation as given in “Appendix 2”):

14 $$\begin{aligned} \lambda_{g} = \mathop \prod \limits_{\alpha = 1}^{N} \sqrt {\lambda_{g,\alpha } } = \mathop \prod \limits_{\alpha = 1}^{N} \exp \left\{ {\frac{k}{{6\beta^{2} }}\left[ {1 - \left( {1 + \beta t} \right)e^{ - \beta t} } \right]D_{g,\alpha } - D^{\text{th}} } \right\}, \end{aligned}$$

with

15 $$\begin{aligned} D_{g,\alpha } = \frac{1}{{r_{f} }}{\text{erf}}\left( {\frac{{\bar{\psi }_{f,\alpha }^{ \hbox{max} } }}{{m_{f} }}} \right). \end{aligned}$$

where $k$ and $\beta$ are the material parameters, and $D^{th}$ is the threshold value for the growth to start. Since the accumulation of neointimal hyperplasia tissue in human peaks at 6 months after stent deployment (Hoffmann and Mintz 2000), a time period of 6 months was chosen for tissue growth in this study. The value of $\beta$ was chosen as 0.05 days^−1^ so that the tissue growth was able to approach its maximum volume over 6 months. The value of *k* was chosen as 0.5 days^−2^ so that the ISR rate for the XIENCE Sierra could reach ~ 20% over 6 months which was close to the clinical follow-up (Serruys et al. 2005; Rizik et al. 2017). Also, it was assumed that the tissue would grow as soon as the damage exists, so $D^{th}$ was taken as 0.

Again, a VUMAT subroutine was coded to realize the growth model in Abaqus/explicit computationally. To verify the subroutine, the simulation of angioplasty in Fereidoonnezhad et al. (2017) was repeated, using exactly the same FE set-up and the models of tissue damage and growth for arterial layers (see “Appendix 1”). Basically, a subroutine was programmed for the same tissue-growth model as that in Fereidoonnezhad et al. (2017), and the simulation results matched the work of Fereidoonnezhad et al. (2017), as shown in Fig. 16 in “Appendix 1”. This independent exercise verified the numerical algorithm used for coding the subroutine. Then, the subroutine was modified by adopting a new growth stretch proposed in this study for the ISR simulation (i.e., Equation (14)).

Continuum-based tissue-growth models, different from the model in Fereidoonnezhad et al. (2017), also exist in the literature. For instance, Boyle et al. (2011, 2013) proposed a mechanobiological model and used a lattice-modelling approach to simulate the development of ISR. The model was able to quantify the volume of neointima hyperplasia and give a quantitative comparison between different stents. Escuer et al. (2019) developed another damage-related volumetric growth model, in terms of densities/concentrations of important species such as growth factors, matrix metalloproteinases, extracellular matrix and contractile and synthetic SMCs. This model was used to simulate the development of ISR, and the results suggested that the arterial wall response was driven by the damage area, proliferation of SMCs and the collagen turnover. In addition, a so-called ghost mesh was used to simulate the neointimal remodelling procedure after stent deployment (Boland et al. 2016, 2019). However, stenting procedure was not simulated due to the complexity of generating the ghost mesh. Instead, the stent was modelled in expanded condition and the artery was expanded by pressure loading. In our study, the model proposed by Fereidoonnezhad et al. (2017) was chosen as it can simulate the tissue-growth behaviour within a finite element framework, thus allowing for a natural integration with the stenting simulation step.

### Material models for stent and balloon

**Fig. 3:**
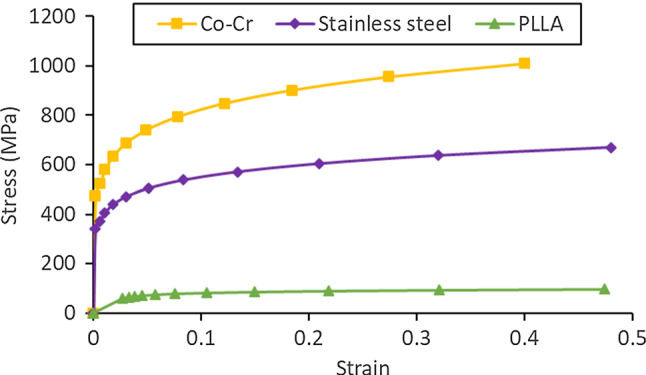
Engineering stress–strain curves of Co–Cr, stainless steel and PLLA

In this paper, XIENCE Sierra (Abbott Vascular, USA), Endeavor (Medtronic Vascular, USA) and ABSORB GT1™ stents (Abbott Vascular, USA) were considered. The XIENCE Sierra and Endeavor stents were made of Co–Cr L605, with a density of 9.12E−9 t/mm^3^, the Young’s modulus of 222,000 MPa and the Poisson’s ratio of 0.29. The plastic behaviour of Co–Cr alloy was provided in Poncin and Proft (2004). A semi-compliant balloon used to inflate the Co–Cr stents was assumed to be made of Pebax 7233, with a density of 1.01E−9 t/mm^3^, the Young’s modulus of 510 MPa and the Poisson’s ratio of 0.30 (MatWeb 2018). The ABSORB stent was made of biodegradable PLLA, with a density of 1.4E−9 t/mm3, the Young’s modulus of 2200 MPa and the Poisson’s ratio of 0.3 (Schiavone et al. 2016). The plastic behaviour of PLLA was described by the stress–strain response measured by Pauck and Reddy (2015). The compliant balloon used to inflate ABSORB was assumed to be made of polyoctanediol-co-citrate (POC), with a density of 1.1E−9 t/mm^3^, the Young’s modulus of 49.79 MPa and the Poisson’s ratio of 0.31 (Ponkala et al. 2012). To investigate the effects of stent materials, a 316L stainless steel was also considered, with a density of 7.95E−9 t/mm^3^, the Young’s modulus of 193,000 MPa and the Poisson’s ratio of 0.27. The plastic stress–strain behaviour was provided in Poncin and Proft (2004). The engineering stress–strain relationships of Co–Cr, stainless steel and PLLA are compared in Fig. 3.

## Finite element simulations of stenting and in-stent restenosis

### Models for artery, plaque, stents and balloon

**Fig. 4:**
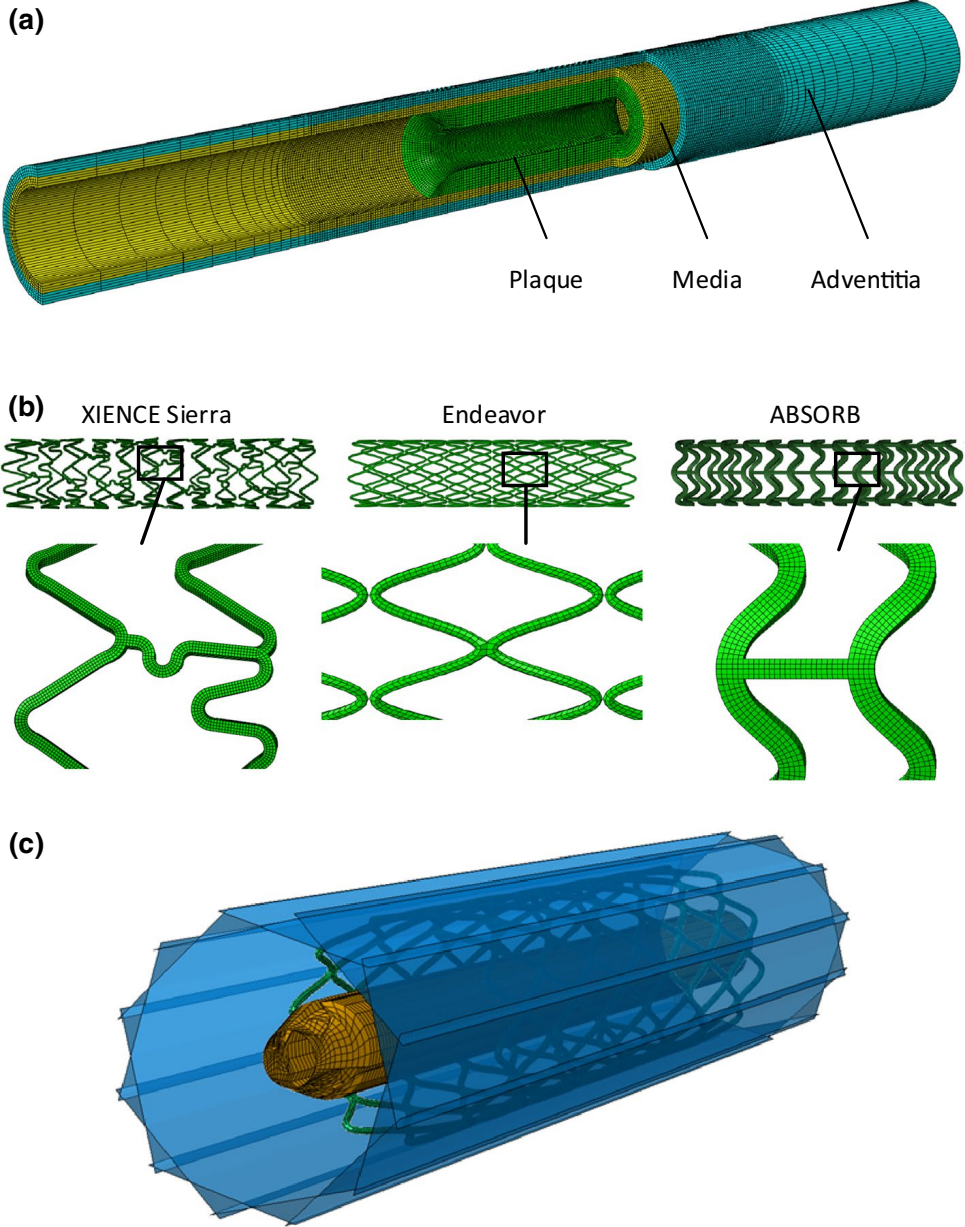
Finite element models: **a** coronary artery-plaque assembly; **b** XIENCE Sierra, Endeavor and ABSORB stents with zoomed-in views; **c** balloon-stent assembly with 12 rigid plates

A two-layer coronary artery (a general coronary artery instead of a specific one), with an overall wall thickness of 0.66 mm, an inner diameter of 3 mm and a length of 40 mm, was modelled. Specifically, the artery consisted of an adventitia and a media layer, with a wall thickness of 0.34 mm and 0.32 mm, respectively (Holzapfel et al. 2005). An extremely thin intima layer was not considered in the simulations due to its negligible contributions to artery deformation. Specifically, for young healthy adults, the intima usually consists of one or two layers of endothelial cells and has a thickness of 2–4 µm (Crawford et al. 2009; the intima thicknesses may increase slightly with age). As discussed in Holzapfel et al. (2005), the intima layer intends to thicken throughout the human life, and the “thickened intima” is believed to be neointima, instead of a healthy intima layer. In our work, the stenotic artery was considered as a healthy artery with accumulated plaque. The plaque was modelled as a symmetric layer inside the artery, with a length of 10 mm and a stenosis of 50% (i.e., an inner diameter of 1.5 mm). Hexahedral elements with reduced integration (C3D8R) were used to mesh the artery and the plaque. It should be noted that Abaqus explicit, used in this work, does not support incompressible hyperelastic materials or hybrid elements, and thus, hybrid elements could not be used here. In the radial direction, the artery was meshed with four rows of elements for each tissue layer, and the plaque was meshed with 8 rows of elements. In the longitudinal direction, a bias control was used to mesh the artery, allowing for a gradual increase in the element size towards both ends of the artery. The FE model for the artery and plaque is shown in Fig. 4a.

FE models for the XIENCE Sierra, Endeavor and ABSORB stents were built using Abaqus CAE as shown in Fig. 4b; all three stents had a length of 12 mm and an outer diameter of 3 mm. The strut thicknesses of the XIENCE Sierra, Endeavor and ABSORB were 81 µm, 91 µm and 150 µm, respectively. The tri-folded balloon, used to inflate the stents, had a length of 16 mm and a diameter of 3.2 mm in a fully inflated shape. NX 8.5 (Siemens PLM Software, UK) was used to create the tri-folded balloon model. Specifically, a series of cross-sectional sketches were created on four uniformly distributed datum planes in fully folded configuration. These cross-sectional sketches were then extruded to produce the 3D geometry by using the sweep tool. Then, the 3D geometry was imported into Abaqus CAE to produce the final geometry of tri-folded balloon model (see further details in Qiu et al. (2018)). The use of tri-folded balloon was also recommended by Bukala et al. (2017) and Geith et al. (2019) in order to obtain reliable results in FE simulations of stent expansion. In Abaqus, C3D8R and M3D4R (three-dimensional 4-node membrane elements with reduced integration) were used to mesh the stents and the balloon, respectively. The stents and the balloons were firstly crimped by 12 rigid plates to fit in the diseased artery as shown in Fig. 4c. Specifically, radial displacements were applied to the rigid plates to crimp the stents, along with the balloon, to an outer diameter of 1.3 mm.

Mesh sensitivity was systematically carried out in our previous studies, in terms of stent deployment (Schiavone 2015; Qiu 2017). For the stent, four different meshes, namely 2 × 2, 2 × 4, 4 × 4 and 6 × 6 layers of elements across the width and thickness, were compared. For the artery, also four different meshes, namely 1, 2, 4 and 6 rings of elements across the wall thickness, were compared for each arterial layer. The simulations showed a good convergence, in terms of stress distribution and maximum/average stress values, for the stent meshed with 4 × 4 layers of elements and the artery layer meshed with 4 rings of elements through the thickness direction. Therefore, in this study, 4 × 4 layers of elements were used for stent strut and four rings of elements were used for the arterial layer. The plaque was meshed with 8 rings of elements, twice those for each arterial layer as recommended in Schiavone (2015). This mesh was sufficiently fine to capture the variation of damage and ISR across the vessel layer (as shown in Figs. 7 and 9).

### Boundary and loading conditions

Both ends of the artery were fully fixed throughout the simulations to reflect the constraints imposed by the human–body environment. The process of stent deployment in the diseased artery consisted of balloon inflation, deflation and removal steps. The inflation step was performed by applying pressure on the inner surface of the balloon. The pressure was increased linearly from 0 to 12 atm (1.216 MPa, within the recommended pressure ranges of all the three stents), with the balloon fixed at both ends. Interactions between the artery, the stent and the balloon were modelled as general hard contacts with a frictional coefficient of 0.25 (Ju et al. 2008). A balanced master–slave weighting and finite-sliding formulation were used for all the contact simulations. For balanced master–slave contact, Abaqus/Explicit calculates the penalty forces twice for surfaces in contact, where the two surfaces act as the master surface in turn for each calculation. The weighted average of the two penalty forces is then applied to the contact interaction. The finite-sliding formulation allows for arbitrary separation, sliding and rotation of the surfaces in contact. Subsequently, the deflation step was modelled by releasing the pressure on the inner surface of the balloon, allowing the expanded stent to recoil freely. Interactions between the stent, balloon and artery were maintained in this step. In the balloon-removal step, contact of the balloon with the stent and artery was removed. In the tissue-growth step (6 months), the media began to grow based on the damage caused by the stent deployment. The stent was not included in this step as the model is unable to simulate the growth of tissue over the stent. All simulations were carried out using the Abaqus explicit solver (Dassault Systèmes 2017). Each of them took 48 cores (Westmere Xeon X5650 CPUs at 2.66 GHz) in the cluster of Loughborough University and took about 4 days to finish.

## Results

### Deformation during stent deployment

**Fig. 5:**
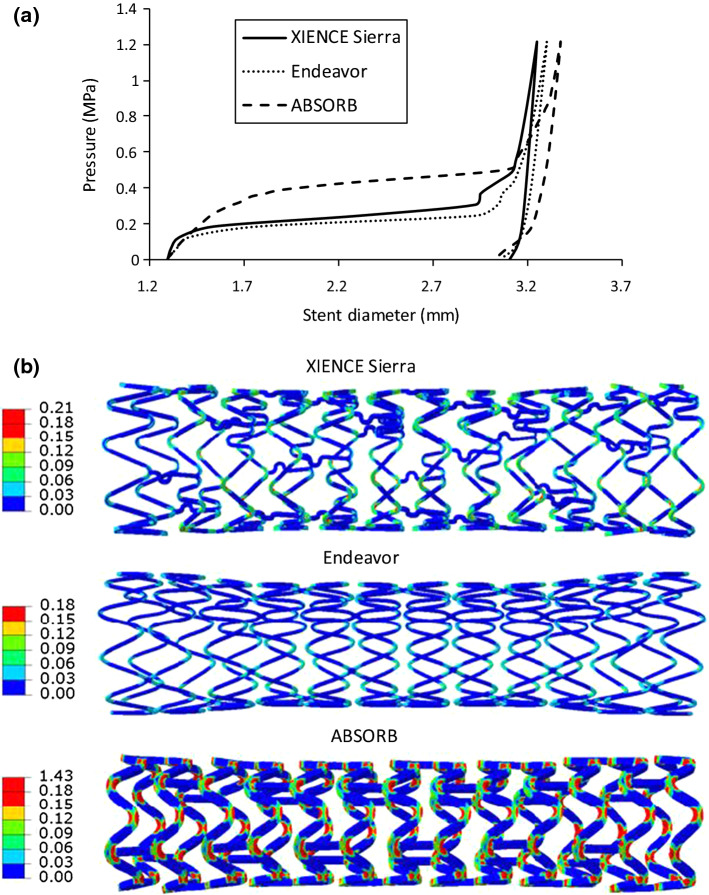
**a** Evolution of outer diameter with pressure for XIENCE Sierra, Endeavor and ABSORB in stenting; **b** equivalent plastic strain in stents after deployment

The outer diameter of the stent was tracked during the stenting process and is plotted in Fig. 5a against the applied pressure. Generally, the stent diameter increased slightly at the beginning, followed by a rapid increase and then a steady state with the further increase in pressure. The three stents experienced different rates of expansion during balloon inflation. The XIENCE Sierra and Endeavor stents showed similar expansion behaviour and reached a steady state at a pressure of around 0.3 MPa, while the ABSORB stent expanded with a lower rate and reached a steady state at a pressure of around 0.5 MPa. The peak diameter achieved by the XIENCE Sierra, Endeavor and ABSORB stents at the maximum inflating pressure was 3.25 mm, 3.30 mm and 3.38 mm, respectively. After the deflation, the stent diameter experienced a gradual decrease, leading to a recoiling effect. The final diameter was 3.11 mm, 3.06 mm and 3.03 mm for the XIENCE Sierra, Endeavor and ABSORB stents, respectively. Clinical trials showed that the lumen gain for the XIENCE stent was greater than that for the Endeavor stent (Serruys et al. 2010), consistent with our results. Also, most ABSORB RCTs (e.g., ABSORB II, ABSORB China, ABSORB Japan, ABSORB III, EVERBIO II) showed less lumen gain for ABSORB stent when compared to metal stents (Rizik et al. 2017), which confirms our results as well. In addition, the equivalent plastic strain in the stents after deployment is plotted in Fig. 5(b). It can be seen that the plastic deformation was dominantly located at the U-bend regions in all three stents. The maximum equivalent plastic strain for the XIENCE Sierra stent was slightly higher than that for the Endeavor stent (i.e., 0.21 for XIENCE Sierra vs. 0.18 for Endeavor), while that in the ABSORB stent was even higher (1.43), reflecting the weak properties for PLLA. All three stents showed dog-boning and asymmetrical cell expansion, which affected the stress/strain peaks and overall expansion as shown in Fig. 5.

**Fig. 6:**
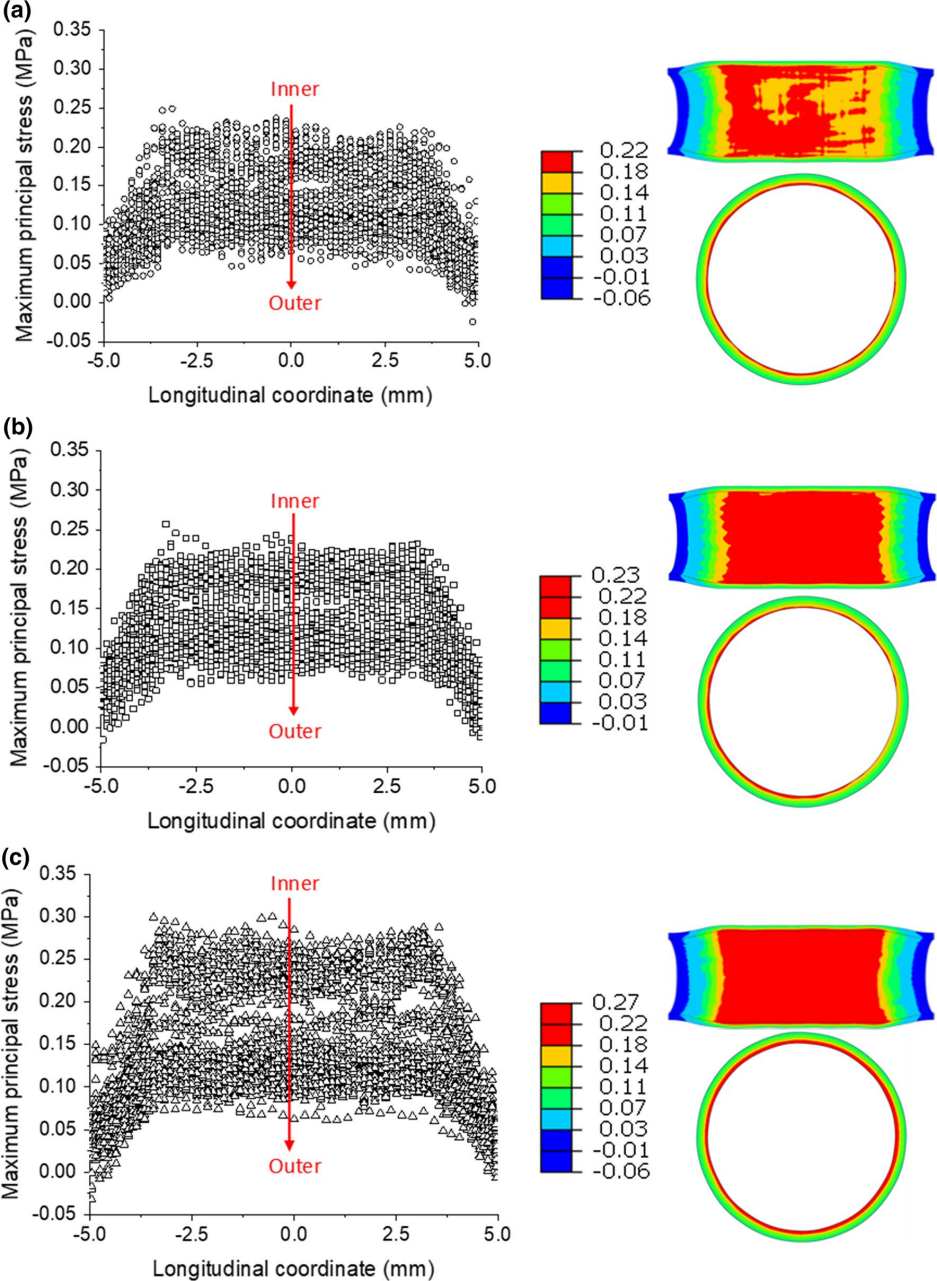
Longitudinal distributions and longitudinal and cross-sectional contour plots of maximum principal stresses (MPa) in the media layer at maximum pressure in deployments of **a** XIENCE Sierra, **b** Endeavor and **c** ABSORB

In addition, the maximum principal stress developed in the media layer at peak inflating pressure is plotted in Fig. 6 against the longitudinal position, where the origin is taken as the plaque’s centre. The symbols are data retrieved from the integration points inside the media layer (also for the rest), which vary across the thickness direction of each layer (inner to outer positions). Corresponding contour plots are also presented to demonstrate the distribution of the maximum principal stress. Here, the results are shown for the media layer only as they are directly related to the tissue growth and development of ISR. The maximum principal stresses decreased from the inner to the outer radii of the media. The stresses in the media were at a similar level for the XIENCE Sierra and Endeavor stents, while higher stresses were noticed for the ABSORB stent due to the larger expansion. Since the plaque thickness decreased towards both ends, the stenting-induced stresses in the media layer also decreased at the ends of the plaque. Similar behaviour was also found for the stresses developed in the plaque and the adventitia layer. The stress distribution in Fig. 6a is asymmetric, mainly due to asymmetrical deformation of the stent. However, the asymmetricity is not significant according to the longitudinal distribution plot (left picture of Fig. 6a).

### Tissue damage and development of in-stent restenosis

**Fig. 7:**
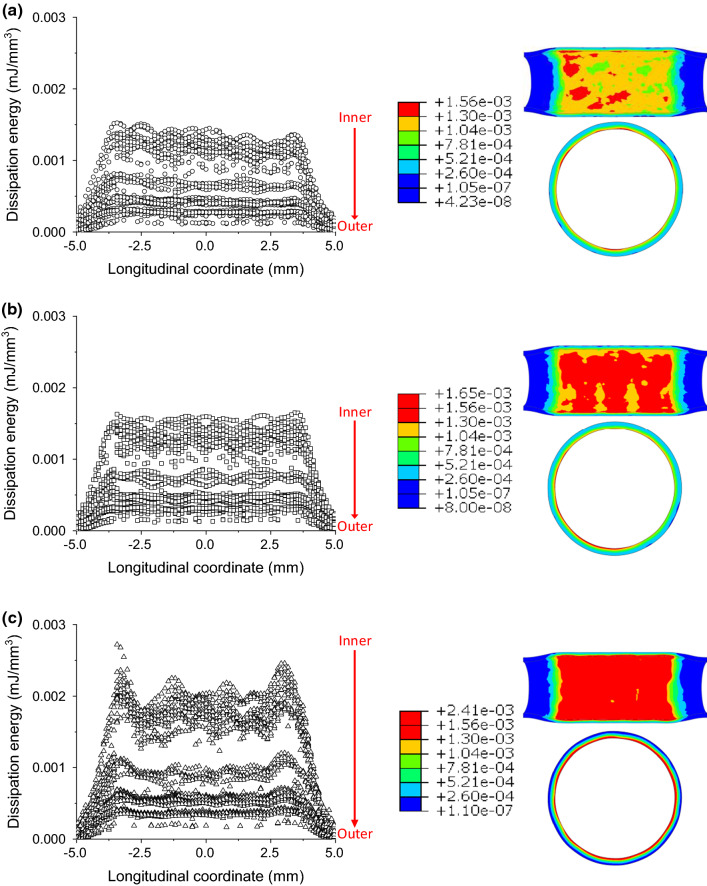
Longitudinal distributions and longitudinal and cross-sectional contour plots of dissipation energy (mJ/mm^3^) in the media layer contributed by damage after deployments of **a** XIENCE Sierra, **b** Endeavor and **c** ABSORB stents

Here, dissipation energy (density) was used to quantify the damage in the tissue caused by stenting. Similarly, the dissipation energy in the media layer after stenting is plotted in Fig. 7 against the longitudinal position, together with the corresponding contour plots. Again, the symbols are data retrieved from the integration points inside the media layer, which vary across the thickness direction of each layer (inner to outer positions). As shown in Fig. 7, the Endeavor stent caused more damage to the media layer than the XIENCE Sierra stent. The ABSORB stent introduced the highest level of damage to the media layer, as indicated by the maximum value of dissipation energy (Fig. 7c), which is about 1.5 times higher than that caused by the two metallic stents (Fig. 7a, b). The points are separated into several distinct groups by dissipation energy. With a closer look, the gaps are also observed for the maximum principal stress plot (Fig. 6), although not as significant as the dissipation energy. This is simply because the hyperelastic behaviour of vessel tissue (i.e., highly nonlinear) and the gaps for the dissipation energy and the maximum principal stress do not follow a linear scaling relationship. Specifically, a small variation in the maximum principal stress can be amplified in the dissipation energy.

**Fig. 8:**
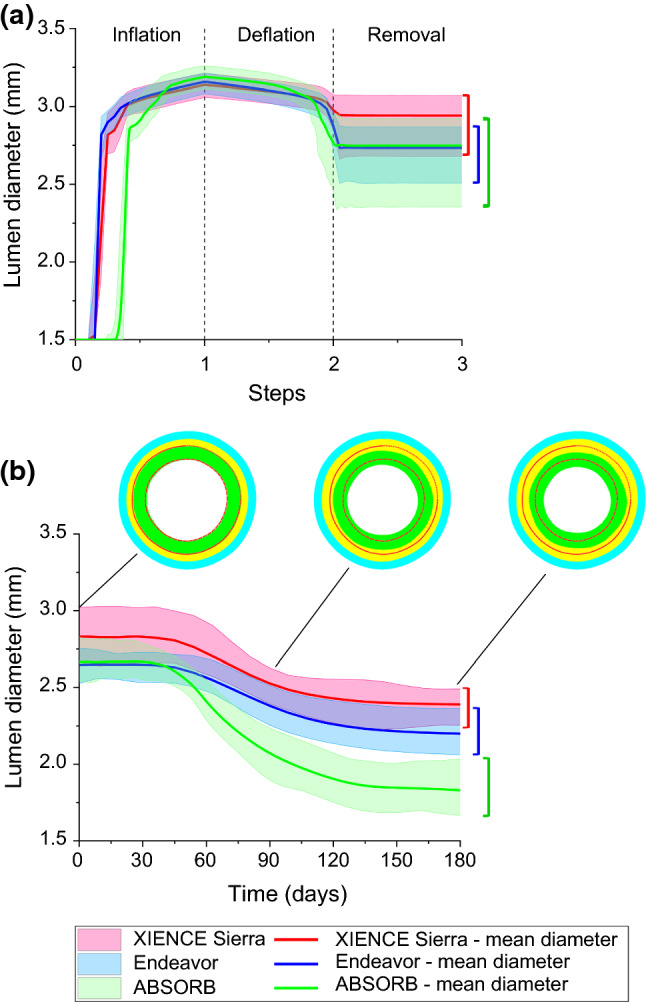
Evolutions of lumen diameter during **a** stenting and **b** tissue growth for XIENCE Sierra, Endeavor and ABSORB stents with cross-sectional views of artery at 0, 90 and 180 days (plaque: green; media layer: yellow; adventitia layer: blue; inner and outer boundaries of plaque: red dotted lines)

**Fig. 9:**
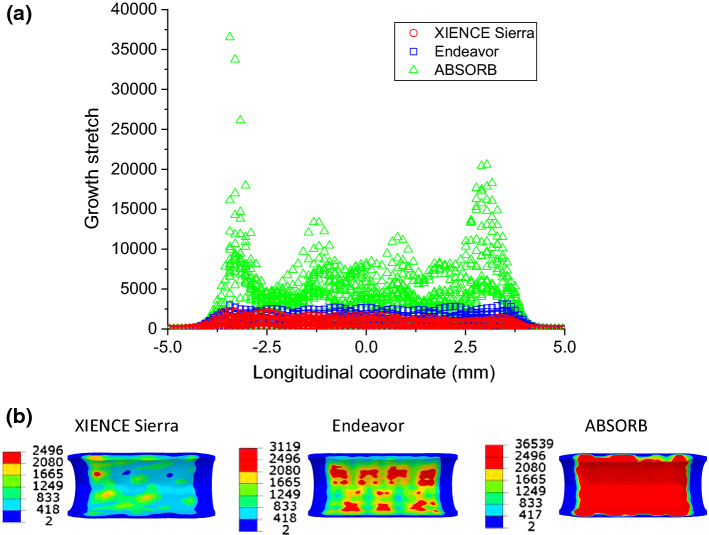
**a** Longitudinal distribution and **b** contour plots of growth stretch $\lambda_{g}$ in the media layer at 6 months after deployment of XIENCE Sierra, Endeavor and ABSORB stents

The change of lumen diameter was adopted to represent the ISR level. This diameter was obtained by tracking and averaging the radial displacements of all the nodes on the inner surface of the plaque. The change of lumen diameter in the stenting and tissue-growth steps for the XIENCE Sierra, Endeavor and ABSORB stents is compared in Fig. 8. The lumen was expanded to a similar diameter for the XIENCE Sierra, Endeavor and ABSORB stents (i.e., 3.14 mm, 3.16 mm and 3.19 mm on average, respectively) at the end of inflation (Fig. 8a). After 6 months of stent implantation, the mean lumen diameter reduced to 2.39 mm, 2.20 mm and 1.83 mm for the XIENCE Sierra, Endeavor and ABSORB stents, respectively (see Fig. 8b). The growth stretch $\lambda_{g}$ of the media layer after 6 months of stent implantation was more localized for the Endeavor stent and had a higher maximum value when compared to that for XIENCE Sierra stent (Fig. 9). For both metallic stents, the growth stretch was concentrated in the area in contact with the U-bends of the stent. Also, for the Endeavor stent, the growth stretch became more significant at the locations where the two U-bends were connected (see red regions for Endeavor in Fig. 9b). For the ABSORB stent, the growth stretch $\lambda_{g}$ of the media layer had a maximum value of 36,539, the highest among the three stents studied and around 15 times that for the two metallic stents.

### Effects of stent materials on tissue damage and in-stent restenosis

**Fig. 10:**
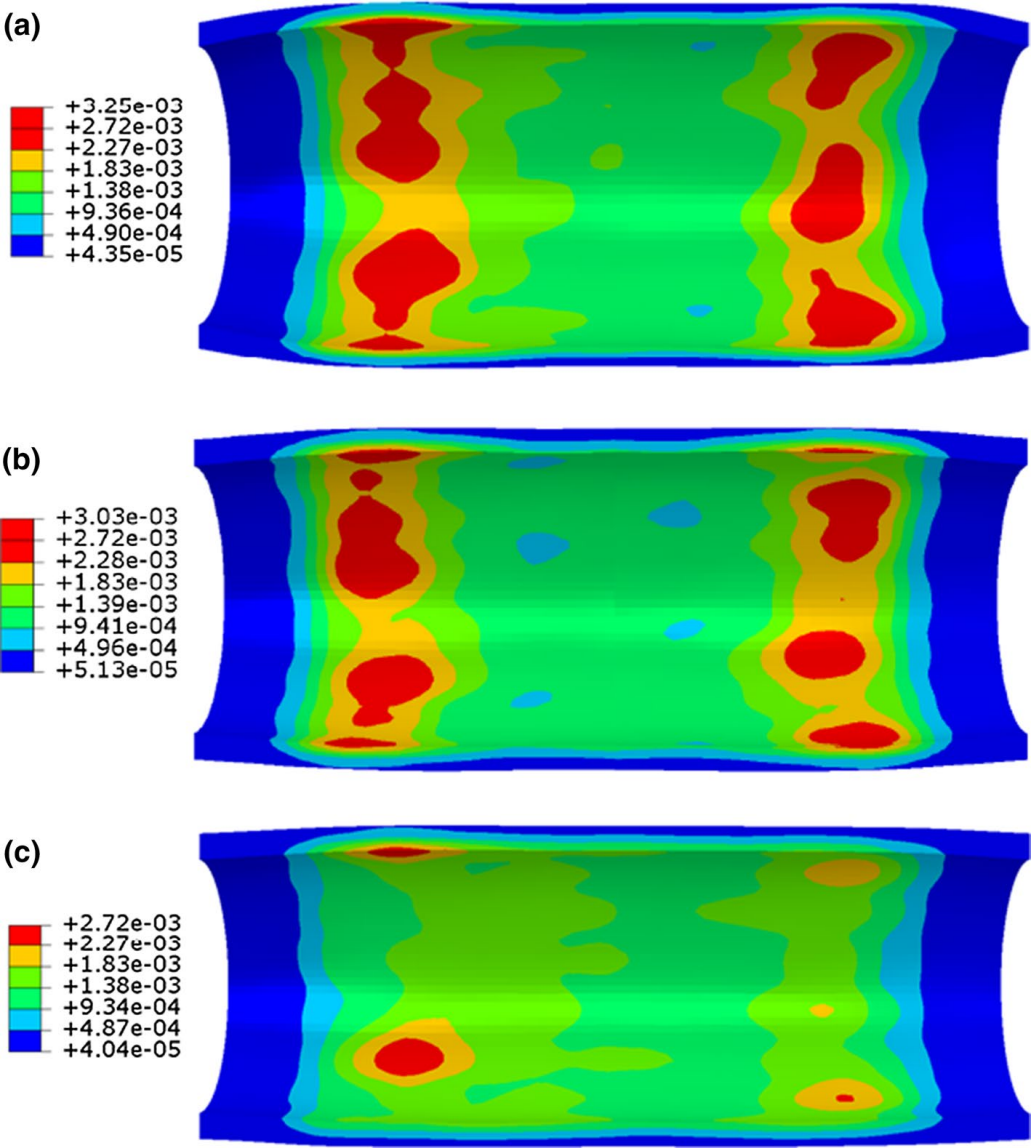
Contour plots of dissipation energy (mJ/mm^3^) in the media layer after deployment of stents made of **a** Co–Cr, **b** stainless steel and **c** PLLA

**Fig. 11:**
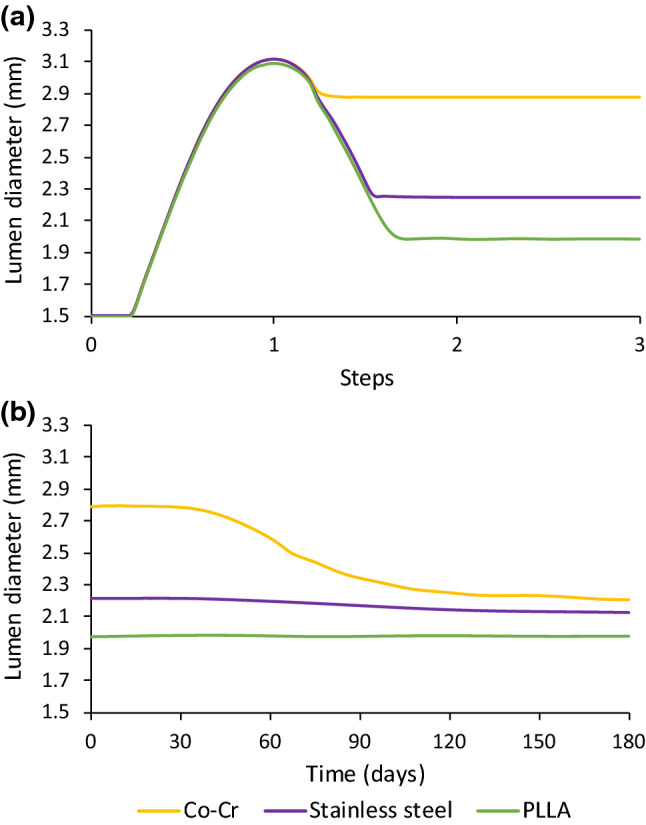
Effects of stent material on in-stent restenosis: evolution of lumen diameter (mean value) during **a** stenting and **b** tissue growth

**Fig. 12:**
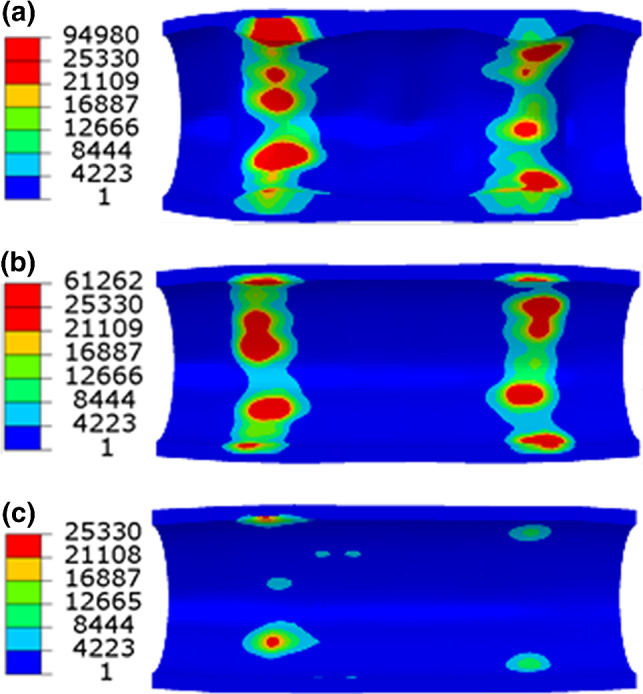
Contour plots of growth stretch $\lambda_{g}$ in the media layer for **a** Co–Cr, **b** stainless steel and **c** PLLA stents at 6 months

As shown in Sects. 4.1 and 4.2, both the stent design and material have an impact on the tissue damage and the ISR rate following stent implantation. To separate these effects, an additional study on the effects of stent materials on ISR was performed by considering a stent with the same design as the XIENCE Sierra stent but made of the Co–Cr alloy, 316L stainless steel and PLLA. In order to achieve the same expansion at the end of inflation, a linear-elastic tube controlled by cosinusoidal velocity was used to expand the stents to the same diameter of 3.0 mm. As shown in Fig. 10, the damage in the media layer appeared to be the least for PLLA and the most for Co–Cr. Since the stainless steel and PLLA stents recoiled much more than the Co–Cr one as shown in Fig. 11(a), the difference of the lumen diameters between day 0 and month 6 was used to compare the severity of the ISR. It can be seen that, for Co–Cr alloy, the ISR was significantly higher than those for the stainless steel and PLLA. Specifically, the rate of ISR was computed as 19.52%, 2.92% and 0.53% for Co–Cr, stainless steel and PLLA, respectively, indicating that Co–Cr caused more severe ISR than stainless steel and PLLA. This was also confirmed by the contour plots of the growth stretch $\lambda_{g}$ in the media at month 6 (see Fig. 12). The maximum value of $\lambda_{g}$ for Co–Cr was 1.55 and 3.75 times higher than those for stainless steel and PLLA, respectively.

### Correlation of artery expansion with in-stent restenosis

**Fig. 13:**
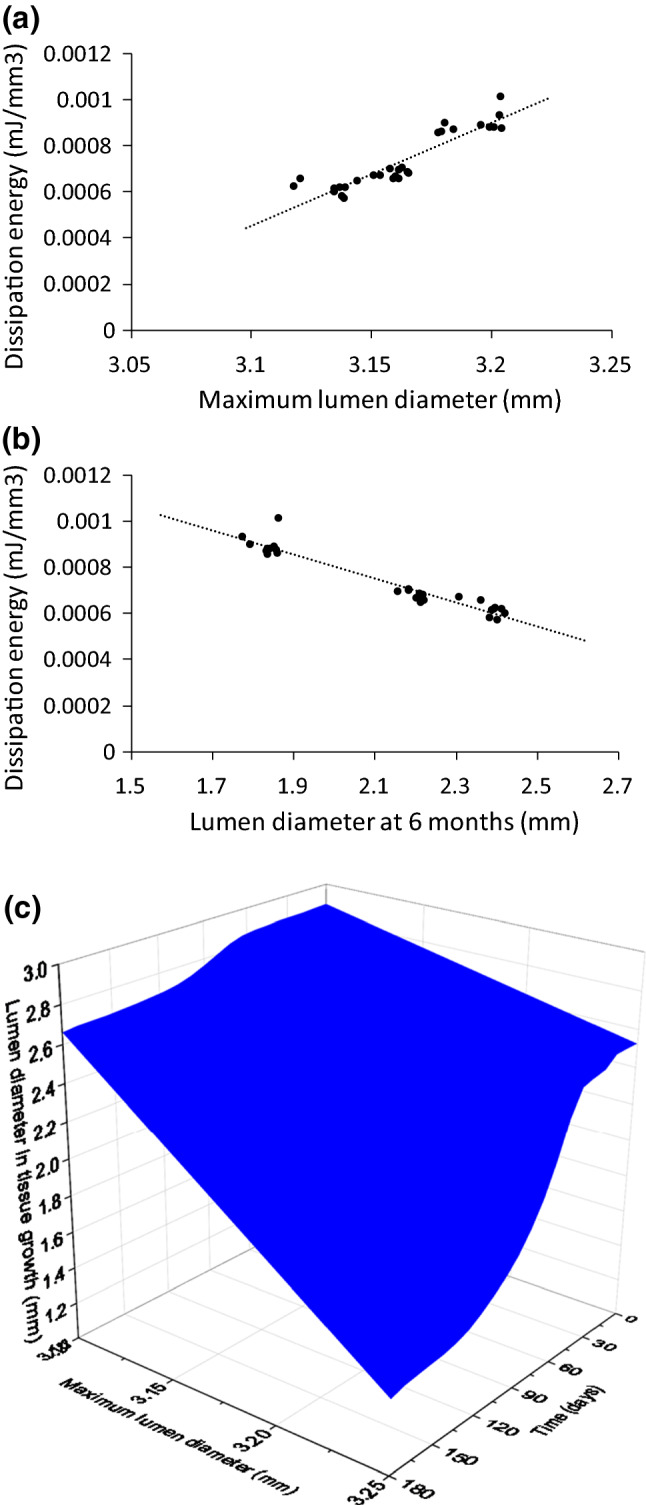
Correlations of tissue damage (dissipation energy) with **a** the maximum lumen diameter and **b** the lumen diameter at 6 months of stent implantation; **c** correlation of in-stent restenosis with the maximum lumen diameter and time

Based on the results above, it seems that the expansion of the artery achieved at peak inflating pressure during stenting determines the amount of tissue damage induced to the media layer, and it was assumed the development of ISR is caused by the tissue damage in the media layer. Here, the dissipation energy of the media layer within the diseased region of the artery was used to represent the amount of tissue damage introduced to the media layer by stenting. The lumen diameter achieved at peak inflating pressure was taken to represent the maximum expansion of the artery during stent implantation, while the lumen diameter at 6 months was used to represent the level of ISR after stent implantation. From the simulations conducted for the XIENCE Sierra, Endeavor and ABSORB stents, data were extracted at ten different sections for each stent. These sections were evenly distributed in the diseased part of the artery along the longitudinal direction. For each section, the value of the dissipation energy was averaged over two interior rings of elements in the media layer, while the lumen diameter was averaged over the nodes on the inner surface of the plaque and shared by the two rings of elements. The extracted lumen diameters at peak inflating pressure and after 6 months of stent deployment are plotted in Fig. 13a, b, respectively, against the extracted dissipation energy in the media layer caused by stenting. Apparently, the dissipation energy in the media layer has a positive linear correlation with the maximum lumen diameter achieved during stent deployment, while it has a negative linear correlation with the lumen diameter after 6 months of stenting. It can be confirmed that an increase in artery expansion causes an increase in damage in the media layer, subsequently leading to the development of more ISR. Additionally, the lumen diameter during the tissue growth is plotted in Fig. 13c as a function of both the maximum lumen diameter and the growth time, showing a direct correlation of the ISR with the maximum lumen diameter over the time after stent implantation. The diagram represents a 3D surface plot of ISR as a function of peak lumen diameter and time, which can be used directly to predict ISR for a given peak lumen diameter and time point. The lumen diameter (on the vertical axis) is negatively correlated with the maximum lumen diameter at 0 days, which is opposed to what one would expect (i.e., a higher initial lumen diameter for the higher maximum inflation diameter). This is because the correlation diagram was constructed from the results obtained using three different stents. For instance, at 0 days, simulations gave lower initial lumen diameter for ABSORB due to more recoiling effect even though its peak lumen diameter was larger when compared to metallic stents. If the same stent was used, higher initial lumen diameter would always correspond to higher maximum inflation diameter.

### Prediction of in-stent restenosis for stent overlapping

**Fig. 14:**
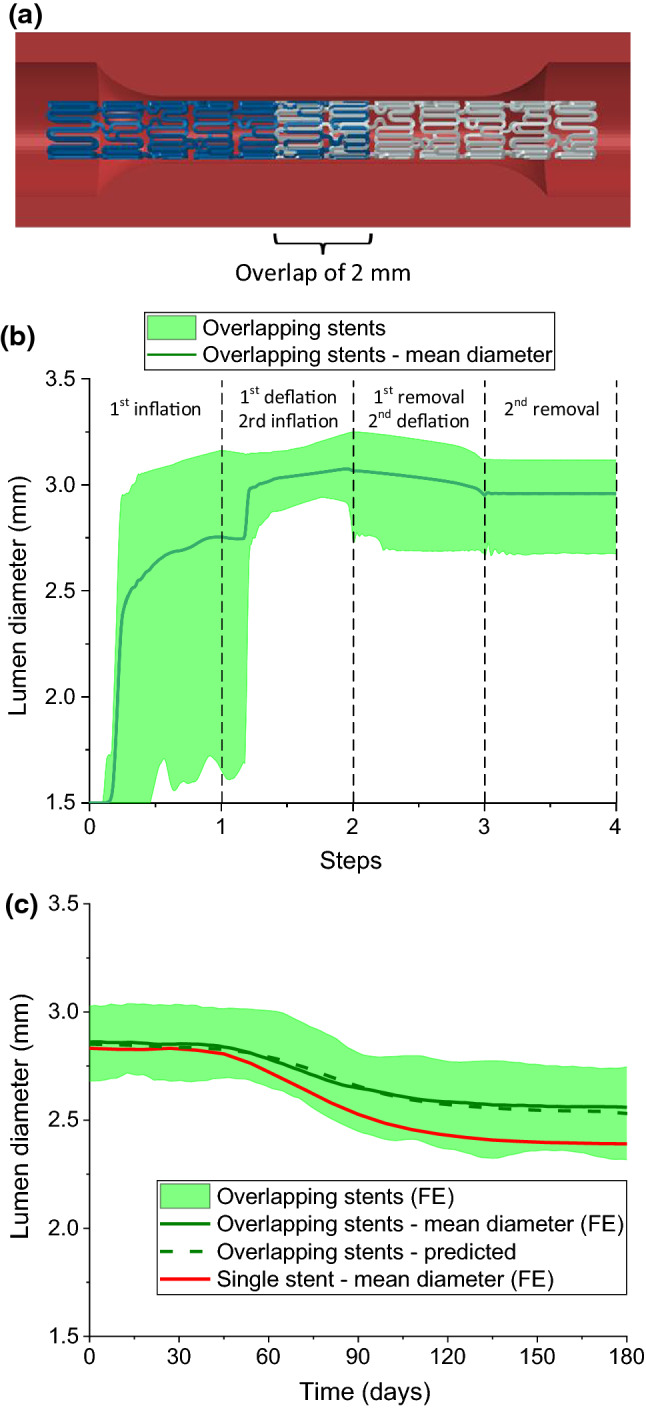
**a** Schematic of artery and two overlapping XIENCE Sierra stents; evolutions of lumen diameter; **b** stenting obtained from FE simulation; **c** tissue growth predicted with correlation diagram in comparison with FE simulations for both overlapping and single stents

In this section, the correlation diagrams presented in Fig. 13 was used to predict the ISR developed after deployment of overlapped stents. For the simulation of overlapped stents, two XIENCE Sierra stents and associated balloons were modelled, with a length of 7 mm and 11 mm, respectively. The stents overlapped by 2 mm as shown in Fig. 14a. During the deployment process, Stent 1 was inflated first and had no interaction with Stent 2. When Stent 1 was in deflation step, the inflation for Stent 2 began and the interaction between the two stents was activated. Evolution of the lumen diameter during stenting is plotted in Fig. 14b, showing the data extracted for all the nodes on the inner surface of the plaque as well as the mean value (line). Substituting the averaged maximum lumen diameter of 3.11 mm into the correlation diagram in Fig. 13c results in the predicted lumen diameter over 6 months of tissue growth, plotted in Fig. 14c as a dashed dark-green line. Simulations were also carried out to evaluate the ISR caused by stent overlapping, and evolution of the lumen diameter during the tissue-growth stage is also plotted in Fig. 14c. Clearly, the prediction and simulations are in a very good agreement. In addition, simulation for a single stent was also carried out and the result is plotted in Fig. 14) as solid red line, indicating the development of more severe ISR for a single stent in this situation.

**Fig. 15:**
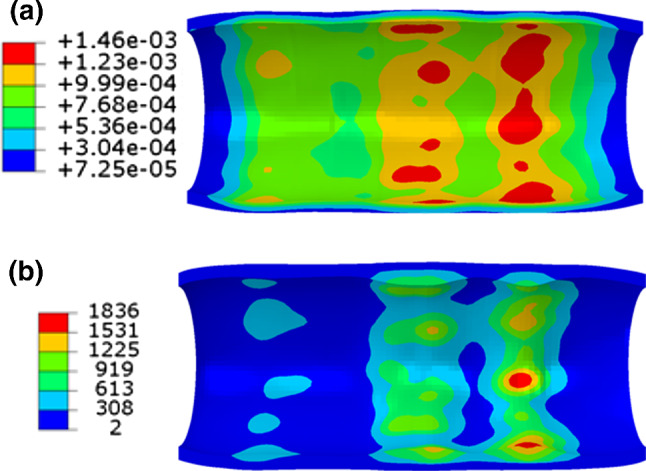
Contour plots of **a** dissipation energy (mJ/mm^3^) in the media layer at peak inflating pressure and **b** growth stretch $\lambda_{g}$ in the media layer at 6 months after deployment of two overlapping XIENCE Sierra stents

**Fig. 16:**
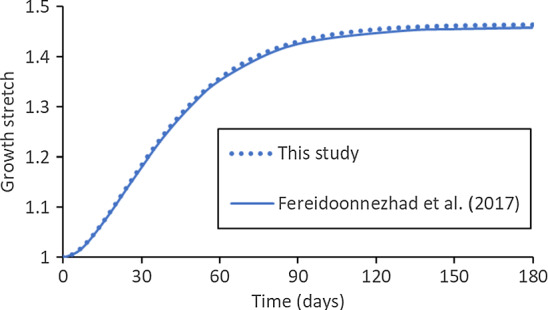
Evolution of growth stretch in intima layer after angioplasty, in comparison with that in Fereidoonnezhad et al. (2017)

Dissipation energy in the media layer obtained from the simulations of overlapping stents is plotted in Fig. 15a. Also, it is seen that they caused less damage in the media layer than a single stent (Fig. 7a). Furthermore, the maximum stenting-induced damage in the media layer was located in the middle region of Stent 2 (Fig. 15a, towards the right end). It should be noted that the two stents were expanded one after the other, instead of simultaneously. Also, both stents were shorter than the plaque and their overlap region was considerably stiffer than a single stent, so it was actually harder to expand two overlapped stents. Therefore, the overall deformation of the artery for the two overlapped stents was less than that for a single stent, causing less damage in the media layer.

## Discussion

This study attempted to quantitatively correlate the stenting-induced damage with the ISR. Clinical studies observed that the major mechanism of ISR was intimal hyperplasia (Hoffmann et al. 1996), a result of proliferation and migration of SMCs from the media layer, distributed over the whole length of the stent. Such proliferation and migration are healing reactions of the vessel wall to the injury of the media layer caused by stenting. Therefore, it is important to understand the stenting-induced damage in the media layer and its contribution to the ISR. Here, a growth model associated with the tissue damage was adopted to describe the tissue-growth behaviour and applied to the medial layer to simulate the narrowing of the vessel after stent implantation. The growth model was based on the multiplicative decomposition of the deformation gradient into an elastic and a growth part, with the latter associated with the geometry changes that the tissues underwent after stenting due to proliferation and migration of SMCs. A connection between ISR and stenting-induced geometry changes was provided by this growth model. It should be noted that the growth part of the deformation gradient describes the increase in the volume of the tissue caused by the proliferation and migration of SMCs. The phenomenon was modelled from a mechanics point of view and unable to model the infiltration of the SMCs into the lumen and the subsequent proliferation. Also, we are not sure how to relate the growth stretch with cell cycle length and population doubling time of SMCs directly. Nevertheless, the simulated rates of ISR were about 20–30% over 180 days, close to those reported in clinical trials that we reviewed in Introduction.

The damage is determined by the maximum strain energy and depends on the loading history. In our simulations, tissue damage, in terms of energy dissipation, accumulated during the balloon inflation or stent expansion. During the subsequent simulation of tissue growth, it was assumed to remain unchanged and no further damage was introduced. So, tissue damage was primarily caused by stenting. Assessment of stenting-induced damage in the arterial wall provided quantifiable and insight information of tissue injury following the stent treatment, aiding a better understanding of ISR development in patients. As demonstrated above, there is a direct correlation between the arterial damage and the ISR. In fact, our results showed that the maximum lumen diameter achieved during the stenting procedure, i.e., the lumen diameter achieved at peak inflating pressure, significantly affected the rate of ISR. These findings are additional to a stress-based damage variable suggested in Zahedmanesh and Lally (2009), highlighting the significance of diameter expansion in ISR. This is also supported by the ISAR-STEREO clinical trial results (Kastrati et al. 2001). Basically, the DS immediately after the procedures showed that the thin-strut group had less optimal lumen gain, but this was found to have significantly reversed at 6-month follow-up (Kastrati et al. 2001).

Furthermore, this study investigated the effects of stent design and materials on ISR using FE simulations. Currently, the XIENCE Sierra and the Endeavor stents are the two most advanced DESs on the market thanks to their loaded drugs and stent designs. However, it was reported that, although they were deployed with similar inflating pressure, the mean DS at 1-year follow-up (21.3%) for the Endeavor stent was greater than that (15.2%) for the XIENCE stent (Akazawa et al. 2014). Hence, it is possible the Endeavor stent tends to result in more severe ISR than the XIENCE stent based on the clinical follow-up, as also confirmed by our simulation results. The Endeavor stent has a more flexible design than the XIENCE Sierra stent, which leads to a larger expansion of the artery under the same inflating pressure (Schiavone et al. 2014), introducing more damage to the media layer and, hence, more severe ISR. The ABSORB stent is at the cutting edge of stent development and aims for complete bioresorption after the implantation. Several RCTs, such as ABSORB II (Serruys et al. 2015), ABSORB III, ABSORB Japan (Kimura et al. 2015), ABSORB China and EVERBIO II (Sabaté et al. 2016), were carried out to study the mid- and long-term safety and performance of ABSORB stents. Rizik et al. (2017) compiled a pooled analysis of these RCTs and reported a significantly increased DS for the ABSORB stent when compared to that for the XIENCE metallic stent. This agrees with our simulation results, and there are two major reasons for this adverse outcome. First, the polymeric ABSORB stent is easier to expand during deployment thanks to its lower mechanical properties, resulting in larger expansion of the artery, more damage to the media layer and increased level of ISR. Second, ABSORB stent has a bulkier strut design, which prolongs the vessel-wall injury and slows down the arterial healing process when compared to stents with thinner struts (Nakazawa et al. 2009).

As another result of this study, diagrams of linear correlations between the stenting-induced tissue damage in the media layer with the deformation of the artery and the level of ISR were produced. These diagrams can be adjusted by employing parameter values for the growth model calibrated against new experiments and used to predict the risk of ISR for patients. Quantifying the vascular damage can be regarded as one of the advantages of computational modelling, which is currently a challenge for in vitro, in vivo and clinical studies. However, parameters, which can be measured physically, may be considered as clinical predictors for the occurrence of ISR, such as the peak lumen diameter suggested in this study. Basically, if the maximum lumen diameter during the stenting procedure can be measured in the clinical setting, it can be used as input for the correlations to obtain the stenting-induced damage in the media layer and, subsequently, the ISR rate. However, in the reports of the several reviewed RCTs, none of them measured the maximum lumen diameter during the stenting procedure. Also, the maximum lumen diameter is closely related to the balloon to artery ratio and inflation pressure, and it is generally accepted that a higher balloon to artery ratio results in higher rates of restenosis. In fact, some animal studies specifically used overinflated balloons to induce restenosis (Iqbal et al. 2016). Carrozza et al. (1992) suggested several predictors for ISR in their study, such as lumen diameter after stenting, but did not consider the maximum lumen diameter during the stenting procedure. It is not clear whether it is due to the limitation of the current medical imaging techniques or a lack of clinical data in correlating the maximum lumen diameter achieved during stenting with the ISR rate. Nevertheless, our results indicated a strong correlation between the artery expansion caused by stenting and the evolution of ISR, which can be considered as a potential predictor for ISR in future clinical studies. Although the maximum lumen diameter was suggested here, it should be noted that the ISR can also be influenced by other factors such as stent type and the target vessel which need to be investigated in future work.

The high elastic modulus gives Co–Cr excellent radial strength when used in stents (Mani et al. 2007). Since the thickness of the struts is a significant concern in stent design as mentioned above, its ability to make ultra-thin struts with increased strength attracts attentions. A 316 stainless steel was the most common-used metal for stents due to its well-suited mechanical properties and excellent corrosion resistance. However, non-MRI compatibility, poor visibility in fluoroscopy and potential allergic reactions limit its clinical usage. Studies showed that vessel healing completed in 3–9 months (Williams and Awan 2017), after which the stents were not needed to hold the vessel any longer. This led to the development of bioresorbable polymeric stents, which were expected to be fully resorbed in a relevant period of time. However, some studies showed that biodegradable polymers induced additional inflammatory reactions. From a mechanics point of view, our study indicated that softer materials such as polymer caused a lower tissue growth of the vessel wall than stiffer ones such as metallic alloys. However, due to the weaker mechanical properties of softer materials, the stent design has to be bulkier in order to achieve sufficient mechanical strength, which overlays the advantage of the material choice and can lead to the development of more severe ISR, as proved by the simulation results in Sects. 4.1 and 4.2. In our opinion, in addition to high biocompatibility, two aspects are critical for a successful bioresorbable stent, i.e., mechanical properties and absorption time. We are aware that ABSORB has been pulled from the market, due to the clinical problems such as the increased incidence of thrombosis associated with the weaker mechanical properties of PLLA. Magmaris scaffold made of magnesium alloy seems to be more successful in terms of mechanical properties, but it degrades too fast. So, for a better bioresorbable stent, it is critical to tailor the degradation rate, as well as control the change of mechanical properties, of bioresorbable stents through novel material processing and optimal structural design, with validation against thorough in vitro and in vivo studies.

According to the manufacturers’ instructions, it is recommended that, for stent deployment, the inflation and deflation of the balloon should be performed within tens of seconds. However, for all simulations in this study, the step time for balloon inflation and deflation was set to be 0.1 s, i.e., significantly shorter than those recommended. This is due to the constraints on the computational time of the simulations. For instance, simulation times would be increased by a factor of 20 if the inflation and deflation times are taken as of 1 s, i.e., an increase of 10 days in actual computing times. This would become unrealistic for the simulations carried out in this study. Still, the simulations for balloon-inflation times of 0.1 s and 1 s were compared, and the results were within 5% difference in terms of the lumen diameter and stress distributions. Another limitation of this study is related to the experimental data used for constitutive-model calibration. The data for the plaque in Maher et al. (2011) were obtained from the unconfined cyclic compression, instead of tension, tests on specimens from the human carotid atherosclerotic plaque, and it is worthwhile to point out that the plaque might behave differently under tension. The parameters for the modified HGO-C model with damage were calibrated against the experimental data for the thoracic aorta instead of the coronary one. Another limitation is that the same balloon and the same pressure were used for all three stents simulated in this study. However, in reality, different pressures might be used for varied stent deigns which should be taken into account when assessing the design effects. These limitations should be noted when interpreting the simulation results presented in this work. In addition, the artery model was idealized with a uniform and symmetric plaque layer, while, in reality, the plaques might be asymmetric, discontinuous and diffused in the lumen. Hence, patient-specific cases should be explored in the future, based on high-resolution medical imaging of actual diseased artery. Also, for ABSORB, the impact of degradation on the vessel was not considered in this study. From the literature, ABSORB stent only begins to degrade six months after it is placed in the artery and is fully dissolved between 2 and 3 years or even beyond (Naseem et al. 2019; Serruys et al. 2014). So, the effect of degradation is expected to be very limited over 6-month period of times. Nevertheless, future work is required in order to understand the combined effects of vessel injury and stent degradation on the development of ISR.

## Conclusions

A tissue-growth model, linking the stent-induced tissue damage and the ISR, was introduced and employed together with the FE approach to simulate the development of ISR after PCI. The numerical simulations carried out in this study demonstrated that the ABSORB stent resulted in more severe ISR than the XIENCE Sierra and Endeavor stents, which was also confirmed by clinical trials. Regarding the materials used for manufacturing stents, the ISR for softer materials was lower than that for harder ones. However, for polymeric stents, such benefit might be cancelled out by the requirement of bulkier stent design to meet the strength requirements. Correlation diagrams that could be potentially used for predicting the ISR were constructed from a series of FE simulations, confirming a direct correlation between the artery expansion and the ISR. Specifically, the maximum lumen diameter achieved during the stenting procedure is linearly correlated with the stenting-induced damage in the media layer and the development of ISR in the vessel, suggesting that it can be considered as a clinical predictor for the occurrence of ISR.

## Appendix 1

A quarter of a three-layer artery model was created, with an inner diameter of 4 mm and a length of 60 mm. The arterial wall had a thickness of 0.75 mm, including the adventitia layer of 0.25 mm, the media layer of 0.375 mm and the intima layer of 0.125 mm. In the radial direction, the intima, media and adventitia layers were meshed with 1, 3 and 2 rows of C3D8R elements, respectively. Three steps were set up for the simulation. In step 1, the artery was expanded by a uniform pressure applied on its inner surface increasing linearly from 0 to 0.04 MPa. In step 2, the deflation was modelled by releasing the pressure on the inner surface of the artery. In step 3, the tissue growth was simulated for all three vessel layers. The model parameters (both tissue damage and growth models) for the three arterial layers were provided in Fereidoonnezhad et al. (2017). The evolution of the growth stretch obtained from this simulation is shown in Fig. 16 for the intima layer after angioplasty, which agrees with the results in Fereidoonnezhad et al. (2017). This verifies our VUMAT subroutine programmed to model the tissue growth after PCI.

## Appendix 2

In Fereidoonnezhad et al. (2017), the continuity equation is expressed as

16 $$\begin{aligned} tr \left( {{\dot{\mathbf{F}}}_{g} {\mathbf{F}}_{g}^{ - 1} } \right) = \frac{{r_{g} }}{{\rho_{g} }} = \frac{{r_{g}^{0} }}{{\rho_{g}^{0} }}. \end{aligned}$$

In our study, it is proposed that

17 $$\begin{aligned} r_{g}^{0} = \frac{1}{2}k\rho_{g}^{0} t\exp \left( { - \beta t} \right)\mathop \sum \limits_{\alpha = 1}^{N} D_{g,\alpha } - D^{\text{th}} . \end{aligned}$$

Substitution of Eqs. (17) and (12) into Eq. (16) leads to

18 $$\begin{aligned} \frac{{3\dot{\lambda }_{g} }}{{\lambda_{g} }} = \frac{1}{2}kt\exp \left( { - \beta t} \right)\mathop \sum \limits_{\alpha = 1}^{N} D_{g,\alpha } - D^{\text{th}} . \end{aligned}$$

By solving the above Eq. (18), the following can be obtained:

19 $$\begin{aligned} \lambda_{g} = \mathop \prod \limits_{\alpha = 1}^{N} \exp \left\{ {\frac{k}{{6\beta^{2} }}\left[ {1 - \left( {1 + \beta t} \right)e^{ - \beta t} } \right]D_{g,\alpha } - D^{\text{th}} } \right\}. \end{aligned}$$

So, the growth stretch $\lambda_{g}$ in our study followed the continuity equation.
